# Arterial CO_2_ pressure changes during hypercapnia are associated with changes in brain parenchymal volume

**DOI:** 10.1186/s41747-020-0144-z

**Published:** 2020-03-09

**Authors:** Lisa A. van der Kleij, Jill B. De Vis, Jeroen de Bresser, Jeroen Hendrikse, Jeroen C. W. Siero

**Affiliations:** 1 Department of Radiology, University Medical Center Utrecht, Utrecht University, Heidelberglaan 100, 3508 GA Utrecht, The Netherlands; 2 National Institute of Neurological Disorders and Stroke, National Institutes of Health, Bethesda, MD USA; 3 Department of Radiology, Leiden University Medical Center, Leiden, The Netherlands; 4 Spinoza Center for Neuroimaging, Amsterdam, The Netherlands

**Keywords:** Brain, Cerebral blood volume, Hypercapnia, Intracranial pressure, Healthy volunteers, Magnetic resonance imaging

## Abstract

The Monro-Kellie hypothesis (MKH) states that volume changes in any intracranial component (blood, brain tissue, cerebrospinal fluid) should be counterbalanced by a co-occurring opposite change to maintain intracranial pressure within the fixed volume of the cranium. In this feasibility study, we investigate the MKH application to structural magnetic resonance imaging (MRI) in observing compensating intracranial volume changes during hypercapnia, which causes an increase in cerebral blood volume. Seven healthy subjects aged from 24 to 64 years (median 32), 4 males and 3 females, underwent a 3-T three-dimensional T1-weighted MRI under normocapnia and under hypercapnia. Intracranial tissue volumes were computed. According to the MKH, the significant increase in measured brain parenchymal volume (median 6.0 mL; interquartile range 4.5, 8.5; *p* = 0.016) during hypercapnia co-occurred with a decrease in intracranial cerebrospinal fluid (median -10.0 mL; interquartile range -13.5, -6.5; *p* = 0.034). These results convey several implications: (i) blood volume changes either caused by disorders, anaesthesia, or medication can affect outcome of brain volumetric studies; (ii) besides probing tissue displacement, this approach may assess the brain cerebrovascular reactivity. Future studies should explore the use of alternative sequences, such as three-dimensional T2-weighted imaging, for improved quantification of hypercapnia-induced volume changes.

## Key points

- This study illustrates the application of the Monro-Kellie hypothesis to structural MRI.
- Hypercapnia induced an increased segmented brain volume and decreased cerebrospinal fluid volume.
- Blood volume changes can affect the results of brain volumetric studies.
- Hypercapnia volume changes could serve as a volumetric measure of cerebrovascular reactivity.

## Background

The cerebral vasculature plays a critical role in maintaining an optimal supply of cerebral blood flow (CBF) to the brain. This is dependent on cerebral autoregulation and cerebrovascular reactivity (CVR). Autoregulation refers to the mechanism to adjust cerebral perfusion pressure in order to maintain a stable cerebral blood flow, whereas CVR is the capacity to change cerebral blood vessel diameter in response to a vasoactive stimulus. Such vasoactive stimuli are lowered pH, which induces vasodilation directly, and CO_2_, which induces vasodilation both directly and indirectly [1].

**Fig. 1:**
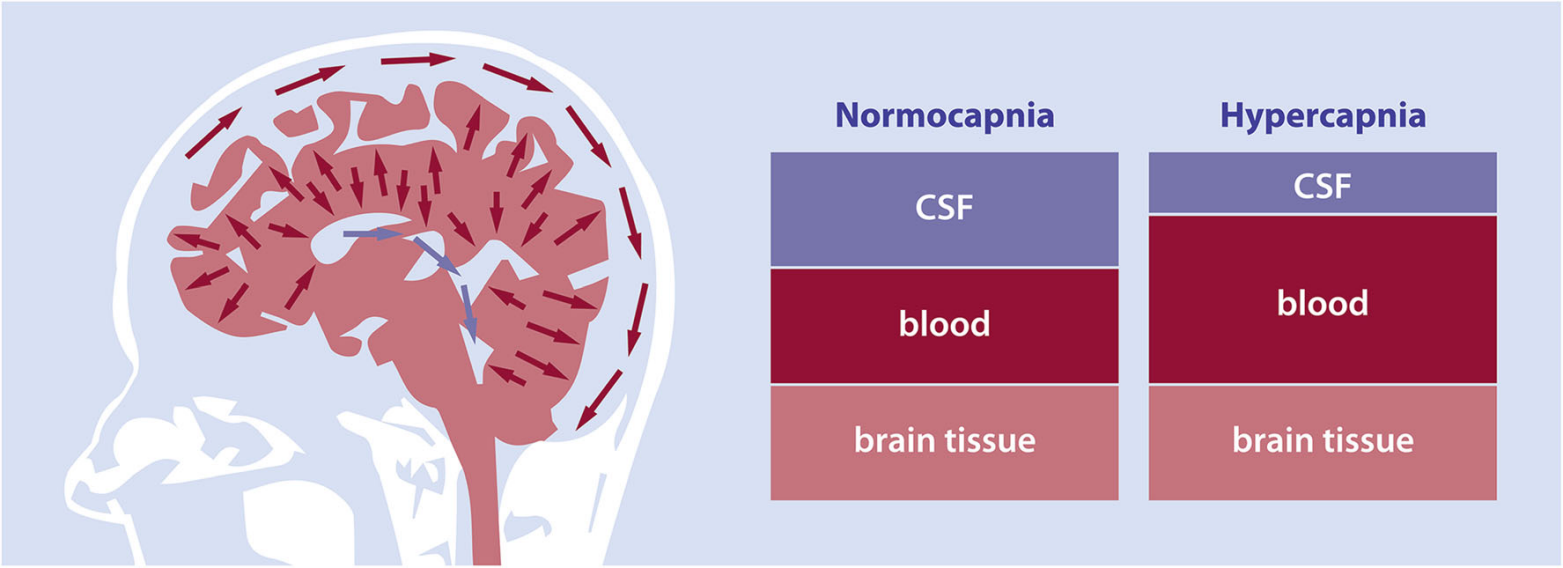
Schematic illustration of the Monro-Kellie doctrine. The Monro-Kellie doctrine describes the dynamic relationship between the intracranial cerebrospinal fluid (CSF), blood, and brain volumes to maintain intracranial pressure. The left panel displays the CSF displacement invoked by an increased cerebral blood volume during hypercapnia. As well, flow in the superior sagittal sinus is shown. Here, CSF is absorbed through the subarachnoid villi. The middle panel is an illustration of the intracranial compartment during normoxic normocapnia. The right panel shows the intracranial volume during hypercapnia, when blood volume increases and CSF volume decreases

Dilation of the cerebral arteries, arterioles and capillaries prompts an increase in cerebral blood volume (CBV) and CBF [2, 3]. An increase in CBV is accompanied by an outflow of cerebrospinal fluid (CSF) to the spinal canal in order to maintain intracranial pressure within the fixed volume formed by the cranium and dura mater as postulated by Burrow’s correction of the Monro-Kellie doctrine (Fig. 1) [4]. As well, CSF can drain to the venous sinuses through the subarachnoid villi. Changes in CBV occur constantly as a response to the cardiac cycle, respiratory cycle, fluctuations in blood gases and locally in response to changes in neural activation [5–7]. CBV also changes in response to respiratory acidosis and metabolic acidosis induced in disease or experimental settings. In experimental settings, the most commonly used vasoactive stimulus is an increase in the partial arterial pressure of CO_2_ (PaCO_2_) by means of a hypercapnic breathing challenge. In these cases, the vasodilatory response is larger than, for example, during the cardiac cycle. Consequently, CBV and CSF volume changes are also expected to be a multitude larger.

The CBV should be taken into account in longitudinal and cross-sectional studies investigating brain volume, as changes in CBV can impact cerebral tissue volume measurements. Intracranial tissue volumes are typically obtained using T1-weighted structural images. Since the longitudinal relaxation times (T1) of grey matter and blood at 3 T are similar [8], blood signals from within grey matter are indiscernible from grey matter tissue signals. Therefore, CBV changes can cause a considerable change in segmented grey matter volume [8–10]. Moreover, a change may occur in partial volume of grey matter relative to the surrounding white matter, which can confound tissue class assignment at tissue borders. As such, changes in (baseline) PaCO_2_ can affect measured tissue volumes on T1-weighted images [10].

Hypercapnia is commonly assumed to predominantly affect grey matter volume measures. Accordingly, observed cortical thickness is increased during hypercapnia [10]. Following the Monro-Kellie hypothesis, we hypothesise that compensating volume changes should be observed in brain parenchymal and CSF volumes on structural magnetic resonance imaging (MRI) in response to cerebral blood volume increases during hypercapnia. In this feasibility study, we investigate the hypothesis application to structural MRI in observing compensating intracranial volume changes during hypercapnia.

## Methods

### Participants

Eight healthy volunteers (median age 29 years, range 24–64 years) were imaged on a 3-T MRI system with an 8-channel head receive coil (Philips Medical Systems, Best, The Netherlands) in August 2015. The study was approved by the Medical Ethical Committee of the University Medical Center Utrecht under protocol number NL39070.041.11. All participants provided written informed consent. Subjects included in this study had two consecutive three-dimensional T1-weighted magnetisation-prepared rapid gradient-echo (MPRAGE) scans as part of their imaging protocol: the first scan was performed during normoxic normocapnia (NC) as a baseline scan and the second scan during normoxic hypercapnia (HC). The subjects were not repositioned between scans. All volunteers completed the scan protocol.

### Imaging protocol

The acquisition parameters of the MPRAGE sequence were as follows: sagittal three-dimensional inversion-recovery turbo field echo; voxel size 1 × 1 × 1 mm^3^, field of view 240 × 240 × 180 mm^3^, matrix size 240 × 240, repetition time 8 ms, echo time 3.2 ms, inversion time 950 ms; flip angle 10°; readout bandwidth 191 Hz per pixel; shot-interval 2100 ms, sensitivity encoding factor 2; and acquisition time 3:11 mins.

### Respiratory challenge

End-tidal O_2_ (EtO_2_) and CO_2_ (EtCO_2_) were controlled using a computer-controlled rebreathing method (RespirAct™, Thornhill Research Inc., Toronto, Canada), which closely match PaCO_2_ and PaO_2_ in healthy volunteers [11]. Subjects were fitted with a sealed mask to prevent air leakage (Tegaderm film, 3 M, Maplewood, MN, USA). Sensor lines connected to the face mask and the monitor provided continuous monitoring of breathing pressure and EtO_2_/EtCO_2_. Before the subjects entered the scanner, a test run was performed in supine position to establish the subject’s baseline EtO_2_, EtCO_2_, tidal volume and respiratory rate. These values were then used as input for the NC and HC blocks. The HC challenge consisted of a boxcar stimulus in which EtCO_2_ was targeted 10 mmHg above a subject’s resting baseline EtCO_2_ over a period of 4:30 min. The MPRAGE scan was started 1 min after the hypercapnic stimulus onset (Additional file 1: Figure S1).

### Image processing

Longitudinal image analysis was performed with the CAT12 toolbox (http://www.neuro.uni-jena.de/cat/) as an extension of SPM12 [12], because of its high accuracy [13] for obtaining grey matter, white matter and CSF volume. Segmentation quality was assessed visually as ‘adequate’ or ‘poor’ (L.A.v.d.K.). The intracranial volume was defined as the sum of CSF, grey matter and white matter volumes and the BPV as the sum of grey matter and white matter volumes. Ventricular volumes were obtained with the region-of-interest CAT12 modality that uses the neuromorphometrics atlas (Neuromorphometrics Inc., http://Neuromorphometrics.com).

### Statistical analysis

Data were given as median and interquartile range (IQR). Statistical analysis was performed with R version 3.4.3 [14]. Absolute volume changes were defined as Vol_HC_ -  Vol_NC_ (mL); relative volume changes were defined as: $\frac{{\mathrm{Vol}}_{\mathrm{HC}}-\kern0.5em {\mathrm{Vol}}_{\mathrm{NC}}}{{\mathrm{Vol}}_{\mathrm{NC}}}\times 100\%.$ Volumes between conditions were compared using a Wilcoxon signed-rank test. A non-parametric test was chosen because of the small sample size.

## Results

Segmentation quality was deemed poor in one out of eight subjects due to excessive motion during hypercapnia. Consequently, seven subjects (four males and three females) were included into the volumetric analysis. The median age of the included patients was 32 years (range 24–64). Baseline characteristics are presented in Table 1. The median mmHg EtCO_2_ increase was 8.68 (IQR 6.77, 9.60) mmHg (*p* = 0.016), and no significant change in EtO_2_ was observed (*p* = 0.156).

**Table 1:** Baseline characteristics and end-tidal CO_2_ values

Subject	Age	Sex	NC EtCO_2_ (mmHg)	HC EtCO_2_ (mmHg)	ΔEtCO_2_ (mmHg)	NC EtO_2_ (mmHg)	HC EtO_2_ (mmHg)	ΔEtO_2_ (mmHg)
1	64	M	36	49	13	113	113	0
2	34	M	32	39	7	111	118	8
3	25	F	33	39	6	111	113	2
4	24	F	36	45	10	107	108	1
5	25	F	37	44	7	107	117	10
6	34	M	28	38	10	119	116	-3
7	32	M	34	42	9	97	109	12
Median			34	42	9^*^	111	113	2^**^
IQR			33	39, 44	7, 10	107, 112	111, 117	0, 9

Values are rounded to the nearest integer. Changes are calculated from unrounded data

*EtCO*_*2*_ End-tidal CO_2,_*EtO*_*2*_ End-tidal O_2_, *NC* Normoxic normocapnia, *HC* Normoxic hypercapnia

^*^p = 0.016, ^**^p = 0.156

**Fig. 2:**
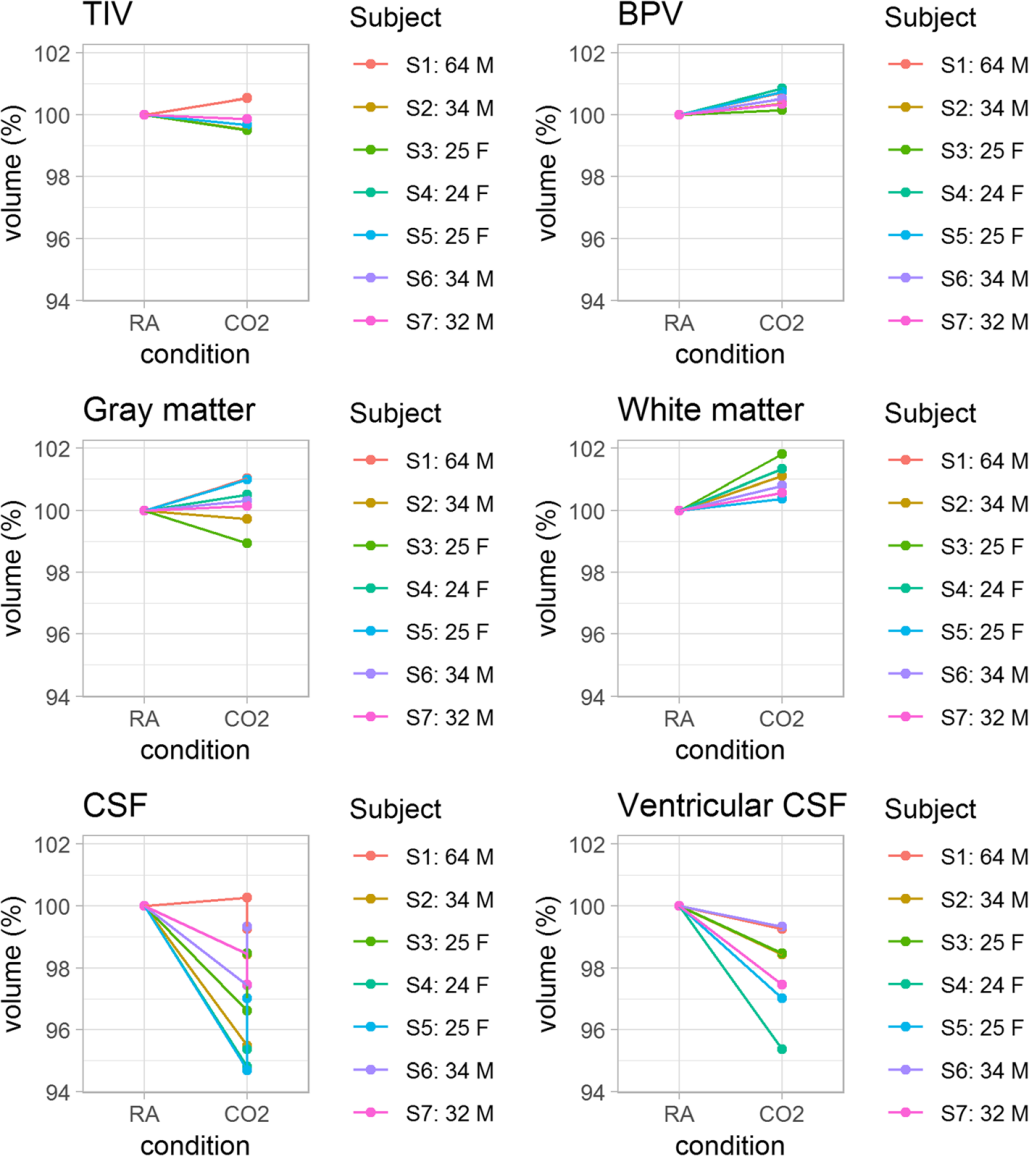
Relative changes in tissue volume (%). Tissue volume changes during hypercapnia are plotted relative to each volume during normocapnia (RA) (set to 100%) per subject. Subject 1 showed an increase in all measured volumes, which resulted in an estimated intracranial volume increase of 9 mL during hypercapnia (top left image). *BPV* Brain parenchymal volume,* CSF* Cerebrospinal fluid, *F* Female, *M* Male, *TIV* Total intracranial volume

Median BPV during NC was 1234 mL (IQR 1162, 1305), which increased by 0.53% (IQR 0.35, 0.72) during HC (*p* = 0.016*;* Table 2). This was an absolute brain volume increase of 6.0 mL (IQR 4.5, 8.5) (*p* = 0.016) (Fig. 1). Grey matter showed a non-significant relative increase of 0.31% (IQR -0.06, 0.75; *p* = 0.469) and an absolute volume increase of 2.0 mL (IQR -0.5, 5.0; *p* = 0.498). In contrast, white matter volume increased by 0.80% (IQR 0.48, 1.21; *p* = 0.016), which is an absolute volume increase of 4.0 mL (IQR 2.5, 7.0; *p* = 0.022) (Fig. 2). A global decrease in CSF volume (3.4%, IQR 2.1, 4.8; *p* = 0.031) was measured with a median change of -10.0 mL (IQR -13.5, -6.5; *p* = 0.034) during hypercapnia compared to normocapnia. As well, a decrease in ventricular volume occurred during HC compared to NC: the median relative volume change was -1.59% (IQR -2.84, -1.14) and the absolute median volume change was -0.38 mL (IQR -0.26, -0.53; *p =* 0.016).

**Table 2:** Absolute volumes under normocapnia and hypercapnia

	Volumes at NC (mL)	Volumes at HC (mL)	ΔVolume (mL)	*p* value
Intracranial volume	1508 (1493, 1587)	1507 (1495, 1581)	-3.0 (-1.5, -6.5)	0.271
BPV	1234 (1162, 1305)	1243 (1167, 1311)	6.0 (4.5, 8.5)	0.016
Grey matter	707 (653, 745)	714 (654, 740)	2.0 (-0.5, 5.0)	0.498
White matter	528 (524, 548)	535 (527, 554)	4.0 (2.5, 7.0)	0.022
Total CSF	295 (279, 318)	285 (267, 312)	-10.0 (-13.5, -6.5)	0.034
Ventricular CSF	17.6 (15.1, 22.3)	17.3 (14.8, 22.0)	-0.4 (-0.3, -0.5)	0.016

Volumes are given as median and interquartile range (in parentheses). Values are rounded to the nearest integer. Volume changes are calculated from unrounded data

*NC* Normocapnia, *HC* Hypercapnia, *BPV* Brain parenchymal volume, *CSF* Cerebrospinal fluid

## Discussion

We found an increase in brain parenchymal volume and a decrease in CSF volume on three-dimensional T1-weighted MRI during hypercapnia. These results demonstrate the feasibility of widely used, structural MRI sequences to observe volumetric reactivity capability of the brain. As well, the results convey several implications for interpreting volumetric MRI results and its potential applications. For instance, diseases and pharmaceutical drugs with vasoactive properties may influence brain and CSF volume assessment. This can affect studies with longitudinal or cross-sectional design such as those regarding atrophy measurements. Also, segmentation analyses of conventional imaging sequences could be used to infer CVR when a vasodilatory stimulus is given.

A median 6 mL increase in BPV was observed during a 9-mmHg hypercapnia stimulus, which is in agreement with an earlier study reporting on ΔCBV as a response to a 10-mmHg hypercapnia stimulus [15]. PaCO_2_ and blood pH effects can occur in various contexts, and our results highlight that they warrant careful consideration in the interpretation and analysis of intracranial volumetric data. An increase in PaCO_2_ and/or a reduction in blood pH can occur in a range of settings aside from a hypercapnic breathing paradigm. As abnormal baseline PaCO_2_ can affect volume measures [10], awareness of its occurrence is important in the abundant longitudinal and cross-sectional studies that investigate brain volume changes. One example of an acute onset respiratory acidosis is the administration of sedative agents in patients undergoing MRI or exercise tasks during MRI [16–18]. In addition, respiratory acidosis can occur in restrictive or obstructive lung disease in which impaired respiratory exchange causes CO_2_ retention, whereas metabolic acidosis may occur in patients with chronic kidney disease due to impaired H^+^ excretion [19, 20]. In cross-sectional studies, differences in PaCO_2_ could confound volumetric MRI results if one group experienced an increased PaCO_2_ while the other group(s) did not. In longitudinal studies, long-term CBV changes related to age and specific pathologies should be regarded [10]. The size of the effects remains to be investigated, but it could have implications for an extensive amount of studies investigating brain volume.

In addition, our approach can be applied as a structural CVR measure. A drawback of our study design is that no data were collected on cerebral blood volume. Therefore, future research is required with CBV measurements along structural CVR to validate the current proof-of-concept study. Structural images are always part of the scan protocol for clinical purposes, and they are readily available even at non-academic hospitals. The 3D T1-based approach offers an excellent contrast between brain parenchyma and CSF. Yet, it also carries the limitation of low contrast to identify the exact spatial boundaries of CSF, dura mater and skull, thereby limiting the method’s ability to detect peripheral CSF volume changes accurately. This makes the sequence suitable for detecting brain volume changes, but not ideal for the detection of total CSF volume changes as indicated by the variability in obtained intracranial volume.

We hypothesised that using high-resolution 3D T1-weighted images would enable us to distinguish volume changes separately for grey matter and white matter. However, the observed volumetric changes during hypercapnia in grey matter and white matter (~ 3 mL) are close to the threshold of volume changes that can reliably be detected with automatic segmentation [21]. As such, we focused on the changes in total brain volume and peripheral CSF. However, for segmenting these larger volumes, 3D T2-weighted sequences are expected to be more accurate and precise than 3D T1-weighted sequences and likely would have been a more appropriate sequence for detecting the hypercapnia-induced larger volume changes. In fact, contrary to T1-weighted images, T2-weighted images carry a high positive contrast between brain tissue and CSF, but also between the peripheral CSF and dura mater and skull. Accordingly, a more reliable measure of total CSF volume and changes therein is expected. Fast 3D T2-weighted sequences are available to determine CSF and brain volume changes [21]. Short imaging time is of particular importance for any sequence used in clinical settings, where CVR assessment can aid in treatment selection and function as a marker of disease severity along with the structural markers [22, 23]. The use of structural images for CVR measurements reduces scan time and cost, because only one additional scan during hypercapnia is appended to the scan protocol.

Limitations of our study are the relatively small sample size and the homogeneous group of healthy subjects. Subsequent studies should elicit whether this method is also applicable for older individuals and for various patient populations. Also, no quantitative T1 measurements were performed in the current study for comparison with morphological changes. While a study with a comparable hypercapnia challenge did observe an increase in grey matter volume using 7-T MRI [10], no significant increase in grey matter volume was found in our study (Additional file 1: Figure S1). In that study, segmentation was performed on “UNI” images (a T1-weighted sequence unaffected by T2*, proton density and field inhomogeneities), proton density and field inhomogeneities. Whereas this does not affect total brain volume, our increase in white matter volume rather than grey matter volume might be explained by these biases. Further, an earlier reported difference in the effect of hypercapnia on T1 in grey matter compared to white matter may contribute to the found changes. A significant decrease in grey matter T1 has been found during hypercapnia, while no significant change occurred in white matter T1 [10]. Therefore, hypercapnia may increase the probability of white matter assignment in voxels at grey-white matter boundaries [24, 25]. Moreover, a previous study showed the dilation of cerebral arteries during hypercapnia [26]. A considerable amount of these arteries are co-located with the segmented white matter. As their signal intensity is closer to white matter than to grey matter, they can contribute to a higher segmented white matter volume during hypercapnia. Future studies should investigate the effect of baseline hemodynamic characteristics on brain volume assessments to ensure that they do not confound brain volumetric studies.

In conclusion, we showed that arterial CO_2_ pressure changes during hypercapnia are associated with changes in BPV and CSF volume in accordance with the Monro-Kellie hypothesis. The found volume changes highlight the relevance of accounting for hemodynamic parameters when interpreting volumetric MRI studies. The proposed volumetric approach to measure CVR may offer a new marker of intracranial tissue reactivity.

## Supplementary information

**Additional file 1: Figure S1.** Hypercapnia challenge (EtCO_2_ on y-axis) and timing of 3DT1-weighted sequence acquisition for Subject 7.

## Data Availability

The datasets used and/or analysed during the current study are available from the corresponding author on reasonable request.

## Abbreviations

CBF: Cerebral blood flow
CBV: Cerebral blood volume
CSF: Cerebrospinal fluid
CVR: Cerebrovascular reactivity
EtCO_2_: End-tidal CO_2_
EtO_2_: End-tidal O_2_
HC: Normoxic hypercapnia
IQR: Interquartile range
MPRAGE: Magnetisation-prepared rapid gradient-echo
NC: Normoxic normocapnia
PaCO_2_: Partial arterial pressure of CO_2_
