# Supplementary material for: Arterial CO2 pressure changes during hypercapnia are associated with changes in brain parenchymal volume
Source: Eur Radiol Exp. 2020 Mar 9;4:17. doi: 10.1186/s41747-020-0144-z (PMC7061094; doi:10.1186/s41747-020-0144-z)
Supplement: Supplementary file 1 — Additional file 1: Figure S1. Hypercapnia challenge (EtCO2 on y-axis) and timing of 3DT1-weighted sequence acquisition for Subject 7. [file 41747_2020_144_MOESM1_ESM.docx]

**Additional file 1**

**
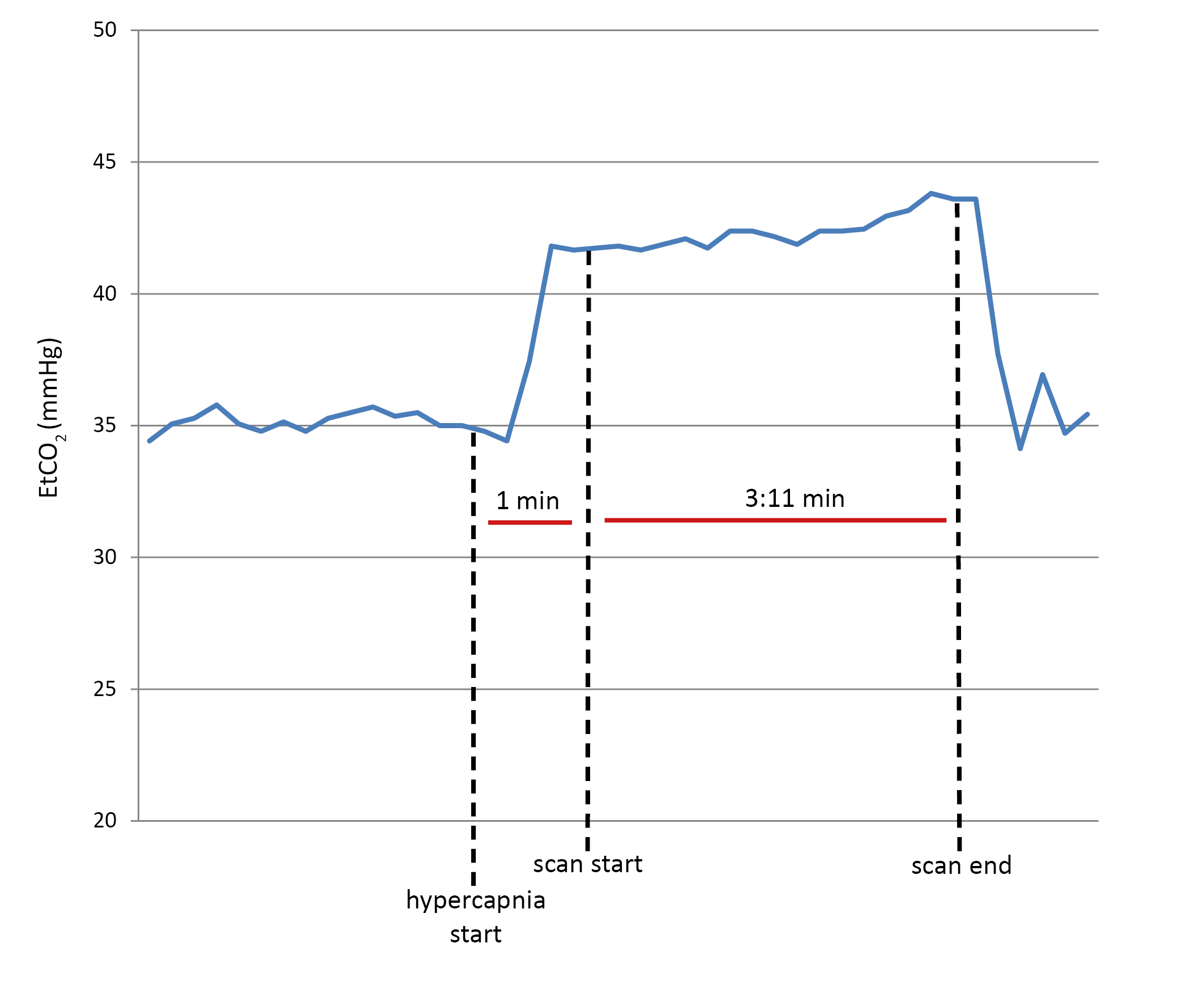
**

**Fig. S1.** Hypercapnia challenge (EtCO_2_ on y-axis) and timing of 3DT1-weighted sequence acquisition for Subject 7.
